# Prevalence and factors associated with hyperglycemia among children living with HIV on dolutegravir in Kabale district, southwestern Uganda

**DOI:** 10.1371/journal.pone.0339268

**Published:** 2025-12-16

**Authors:** Liban Hussein Idow, Timothy Nduhukire, Siyadora Ankunda, Sam Tumwesigire, Filbert Nyeko, James K. Tumwine

**Affiliations:** 1 Department of Paediatrics and Child Health, Kabale University School of Medicine, Kabale, Uganda; 2 Paediatric Haemato-Oncology, Mbarara Regional Referral Hospital, Mbarara, Uganda; 3 Kabale Regional Referral Hospital, Kabale, Uganda; University of Coimbra: Universidade de Coimbra, PORTUGAL

## Abstract

**Background:**

Undiagnosed and poorly managed high blood sugar levels can result in short- and long-term complications in children. Following worldwide introduction of dolutegravir based antiretroviral treatment in 2018, several studies in adult people living with HIV have reported high prevalence of hyperglycemia. Little is known about hyperglycemia in children living with HIV on dolutegravir treatment combinations in Uganda.

**Objectives:**

To determine the prevalence and factors associated with hyperglycemia in children living with HIV on dolutegravir in Kabale district, southwestern Uganda.

**Methods:**

In a cross-sectional study, 271 HIV infected children on dolutegravir (DTG) were recruited using proportional to size and simple random sampling methods from 18 health facilities that provide Anti-retroviral therapy (ART) services in Kabale district. HIV infected children aged below 18 years were considered eligible to participate in the study. Data was collected using structured questionnaires. Blood samples were taken for random blood sugar and HbA1C. Data was analyzed using STATA 17.0 for both descriptive and inferential statistics. Logistic regression analysis was done, crude and adjusted odds ratios obtained.

**Results:**

The prevalence of hyperglycemia was 20.3% (95% CI; 15.9–25.5): Of the children with hyperglycemia, 76% (42/55) had prediabetes and 24% (13/55) had diabetes mellitus.

Red meat consumption (AOR:3.52; 95% CI: 1.4–9.0, p = 0.009) and being above 13 years of age (AOR:2.61; 95% CI 1.4–4.87, p = 0.003) were independently associated with hyperglycemia.

**Conclusion:**

One in every five children with HIV on DTG had hyperglycemia. Among those with hyperglycemia, 24% had diabetes mellitus. Hyperglycemia was more likely to be present in adolescents and those who consumed red meat. Regular monitoring of blood sugar in children living with HIV on DTG is recommended.

## Introduction

Human Immunodeficiency Virus (HIV) is a chronic infection affecting about 1.4 million children worldwide. Of these, more than 80% are in sub-Saharan Africa [1]. In accordance with the World Health Organization recommendation of 2018, the Ministry of Health of Uganda introduced dolutegravir based antiretroviral drugs as treatment of choice for people living with HIV including children [2].

Dolutegravir (DTG) is a 2^nd^ generation HIV integrase inhibitor, which has revolutionalised HIV care [3]. However, several studies mainly in adults have reported new onset hyperglycemia among HIV infected patients following the initiation on DTG [4]. In a study by Gizamba [5], in South Africa, the prevalence of hyperglycemia among HIV infected patients started on DTG was 6.1%. In Uganda, a study involving adult patients living with HIV on dolutegravir in an urban setting reported a 12.8% prevalence of hyperglycemia [6].

Predictors of hyperglycemia among adults living with HIV on dolutegravir include, age, family history of diabetes, obesity, and other comorbidities such as hypertension [6,7].

In Uganda, routine blood sugar monitoring is mainly considered among adults living with HIV on DTG, but is not done for children [8]. There is limited information about the burden and factors associated with hyperglycemia among HIV infected children on dolutegravir. Therefore, we studied the prevalence and factors associated with hyperglycemia in children living with HIV on dolutegravir in Kabale district, southwestern Uganda.

## Materials and methods

### Study design

An analytical cross-sectional study was done to determine the prevalence and factors associated with hyperglycemia among HIV infected children on dolutegravir based anti-retroviral treatment at health facilities in Kabale district, southwest Uganda. The study was conducted from 28^th^ March to 25^th^ May, 2025.

### Study setting

Kabale is one of the districts in southwestern Uganda. It is located approximately 410 kilometers from the capital city Kampala, and hosts Kabale Regional Referral Hospital, which is also the main teaching hospital for Kabale University School of Medicine (KABSOM). The study was done at 18 health facilities; three hospitals and 15 lower health facilities including, health center IIIs and IVs, that provide HIV prevention and treatment services in Kabale district.

### Study population

Children aged < 18 years with HIV infection on dolutegravir based treatment were included in the study. The caregivers of the children aged < 8 years gave written informed consent and those ≥8years to less than 18 years, in addition to the caregivers’/ guardians’ written informed consent, they also provided assent to participate in the study. HIV infected children who were diagnosed with hyperglycemia or diabetes mellitus before being initiated on dolutegravir based treatment were excluded.

### Sample size determination

The sample size for the prevalence of hyperglycemia was calculated using Kish Leslie formula [9] as shown: ($N=\frac{z^{2}{\left(1-P\right)}^{P}}{d^{2}}$); with 20% prevalence from a preprint study in Uganda [10]. The calculated sample size with 95% confidence interval, and 5% precision was 246. Correcting for 10% nonresponse, the final sample size to determine the prevalence of hyperglycemia among HIV infected children was 271.

The sample size for factors associated with hyperglycemia was calculated using Fleiss formula [11] in OpenEpi [12], considering duration on anti-retroviral treatment as an independent factor with an odds ratio of 7.0; 80% power, and 95% confidence interval. The calculated sample size for associated factors, with 10% nonresponse, was 163. This was smaller than the sample size of 271 for the prevalence. Therefore, the sample size of 271 was taken for the study.

### Sampling procedures

There were 441 children living with HIV on dolutegravir receiving care at 18 health facilities in Kabale district. Using proportional to size sampling method, we recruited 271(61.45%) of the 441 children. Simple random sampling was then used to enroll the study participants from each health facility. At each health facility, the eligible participants were identified and assigned with unique numbers. Then, these were written on identical slips which were folded and put into a box. Thereafter, the slips were mixed thoroughly and randomly picked until the required sample was achieved.

### Study variables

The dependent variable in this study was hyperglycemia. The diagnosis of hyperglycemia was based on a random blood sugar of ≥7.8 mmol/L or HbA1C of ≥5.7% [13].

The independent variables were sociodemographic factors including child’s age in years, sex, education status and caregiver factors such as relationships to the child, maternal HIV and paternal HIV status. Child medical factors included duration of dolutegravir based treatment, viral suppression status, HIV AIDS stage, and adherence. In addition, child’s dietary factors were assessed using 24-hour dietary recall.

### Data collection procedures

Interviewer administered questionnaires were used for data collection. The data was collected by the principal investigator with the help of trained research assistants including a nurse/ counselor and laboratory technician from each health facility. A venous blood sample was drawn from each study participant for random blood sugar and HbA1C. Oncall plus glucometer (manufactured by Acon laboratories, San Diego, USA) was used to determine the random blood glucose levels and whole blood samples were tested for HbA1C percentage using Getein 1100 Immunofluorescence quantitative analyzer machine (manufactured by Getein Biotech incorporated, Nanjing, China) at the Rugarama Hospital laboratory. The random blood sugar tests were run immediately while blood samples for the HbA1C were tested within 6 hours of collection. Within 24 hours of blood sample collection, every 30^th^ blood sample was taken to the Lancet Clinical Laboratory (accredited laboratory by KENAS with ML/122, ISO 15189:2012) in Mbarara, Uganda, for quality control of the HbA1C results.

### Data management and statistical analysis

The data collection tools were coded with serial numbers for identification and tracking. Data entry was done using Microsoft excel 2021 for cleaning and storage. A clean dataset was imported into STATA version 17.0 for analysis. Descriptive and inferential analysis were performed. Frequency tables and proportions were summarized with categorical variables. The data was described using means and standard deviations for continuous normally distributed data; and medians with interquartile ranges for skewed data. The prevalence of hyperglycemia was calculated a percentage of children living with HIV on DTG based therapy who had hyperglycemia. At bivariable level, the Chi-square test was used for categorized data to determine the factors associated with hyperglycemia. Fisher’s exact test was used for variables with counts less than 5 in any cell of the tables. Factors with a p value ≤0.2 were taken for multivariable analysis. Multivariable analysis identified factors independently associated with hyperglycemia among HIV infected children using adjusted odds ratios, and 95% confidence intervals; and p < 0.05.

### Ethical considerations

Ethical clearance was obtained from Kabale University Research Ethics Committee (KABREC): reference number KABREC-2025–814. We obtained administrative clearance from Kabale district health office and Kabale Regional Referral Hospital (KRRH). Written informed consent was obtained from the caregivers of children aged below 8 years and informed consent from caregivers and assent from the children aged 8 years and above. The results were shared with the caregivers and patients as well the clinical team for patient care.

## Results

### Baseline characteristics of the study participants

In this study, 271 children living with HIV on dolutegravir based anti-retroviral treatment at 18 health facilities in Kabale district were recruited. The median age of the children was 13 years (IQR:9,15) and 50.55% were female. The median duration of treatment with DTG based regimen was 46 months (IQR: 26, 50). More than half of the children were from urban residences (Table 1).

**Table 1:** Sociodemographic characteristics of HIV infected children on DTG in Kabale district.

Variable	Total N = 271 n		Percentage
Age(years)	0-5	29	10.70
	6-11	78	28.78
	12-17	164	60.52
Gender	Male	134	49.45
	Female	137	50.55
Education	≤Primary	223	82.29
	Secondary	48	17.71
Address	Urban	145	53.51
	Rural	126	46.49
Nutritional status	Normal	209	77.12
	Underweight	30	11.07
	Overweight	32	11.81
Advanced HIV/AIDS	Yes	46	16.97
	No	225	83.03
Viral suppression	Yes	221	81.55
	No	50	18.45
Duration on DTG (months)	≤ 46	141	52.03
	>46	130	47.97
Family history of diabetes mellitus	Yes	7	2.58
	No	264	97.42
Testing for blood sugar before DTG	Yes	16	5.90
	No	255	94.10
Diet type (24 recall)- Carbohydrate	Yes	232	85.61
	No	39	14.39
Red meat	Yes	22	8.12
	No	249	91.88

### Prevalence of hyperglycemia among HIV infected children on dolutegravir in Kabale District

The prevalence of hyperglycemia among HIV infected children on DTG in Kabale district was 20.3% (55/271), 95% CI:15.9–25.5. Of these, 76% (42/55) had prediabetes and 24% (13/55) had diabetes mellitus.

Of the cases with hyperglycemia, 6.64% (18/271), 95% CI: 4–10, had abnormal random blood sugar (RBS) results. However, all the cases with hyperglycemia (55/271) were diagnosed using HbA1c including those with elevated RBS.

### Factors associated with hyperglycemia in the bivariable analysis

Table 2 shows results of bivariable analysis. Children above 13 years of age (OR: 2.65), residing in urban settings (OR: 1.85), and those who had red meat in their last 24 hours (OR: 3.8) were likely to be hyperglycemic. Caregivers’ occupation was associated with less odds of hyperglycemia with p value <0.2. The factors with p value <0.2 were taken into the multivariable regression model.

**Table 2:** Factors associated with hyperglycemia among HIV infected children on DTG in Kabale district (Bivariable analysis).

Variable	Total N = 271	Hyperglycemia n (%)	No Hyperglycemia n (%)	Crude odds ratio, 95% CI	P value
Child’s age(years)					
≤ 13	71	22 (40.00)	138 (63.89)	1	
> 13	200	33(60.00)	78(36.11)	2.65(1.45-4.868)	**0.002**
Gender					
Male	134	23 (41.82)	111 (51.39)	1	
Female	137	32(58.18)	105(48.61)	1.47(0.80-2.67)	0.206
Address					
Rural	126	19(34.55)	107(49.54)	1	
Urban	145	36(65.45)	109(50.46)	1.85(1.00-3.44)	**0.048**
Child’s nutritional status					
Overweight	32	5(9.09)	27 (12.50)	0.7(.2565- 1.91)	
Others	239	50 (90.91)	189 (87.50)	1	0.486
Duration on DTG (months)					
≤ 46	141	26(47.27)	115(53.24)	1	
> 46	130	29(52.73)	101 (46.76)	1.26(0.701-2.29)	0.430
Viral suppression					
Yes	221	43(78.18)	178(82.41)	.764(.3688-1.586)	0.472
No	50	12(21.82)	38(17.59)	1	
Family history of Diabetes mellitus					
Yes		1(1.8)	6(2.8)	0.649(.0138- 5.525)	1.0000
No		54(98.2)	210(97.2)	1	
Advanced HIV AIDS					
Yes	46	11(20.00)	35(16.20)	1.29(0.608-2.74)	0.504
No	225	44(80.00)	181(83.80)	1	
Caregiver’s occupation					
Peasant	178	30(54.55)	148(68.52)	0.551(.30-1.0)	**0.053**
Others	93	25(45.45)	68(31.48)	1	
Child’s diet (24 recall)					
Carbohydrate					
Yes	232	45(81.82)	187(86.57)	0.697(0.317- 1.536)	0.372
No	39	10(18.18)	29(13.43)	1	
Red meat					
Yes	22	10 (18.18)	12 (5.56)	3.8(1.537-9.28)	**0.004**
No	249	45(81.82)	204(94.44)	1	

### Factors associated with hyperglycemia in the multivariable analysis

In the multivariable analysis, a child’s age > 13 years (AOR: 2.61, 95%CI; 1.4–4.87, p = 0.003) and red meat consumption (AOR: 3.52, 95%CI;1.4–9.0, p = 0.00) in the last 24 hours were independently associated with hyperglycemia among HIV infected children on dolutegravir in Kabale district (Table 3).

**Table 3:** Factors associated with hyperglycemia among HIV infected children on DTG in Kabale District, on multivariable analysis.

Variable	Crude odds ratio, 95%CI	P value	Adjusted odds ratio, 95%CI	P value
Child’s age(years)				
≤ 13		1		
> 13	2.65(1.45 −4.868)	0.002	2.61(1.399-4.866)	**0.003**
Address				
Rural	1			
Urban	1.85(1.00-3.44)	0.048	1.43(0.76- 2.70)	0.269
Caregiver’s occupation				
Peasant	0.551(.30-1.0)	0.053	0.631(.332-1.20)	0.161
Others	1			
Red meat consumption				
Yes	3.8(1.537-9.28)	0.004	3.52(1.4-9.0)	**0.009**
No	1			

## Discussion

In this study we aimed to determine the prevalence and factors associated with hyperglycemia among HIV infected children on dolutegravir in Kabale district in southwest Uganda.

The prevalence of hyperglycemia was (55/271) 20.3%. Of the 55 children with hyperglycemia, 42(76%) were prediabetic and 13(24%) had DM.

A similar prevalence of hyperglycemia was reported in a study of children in Uganda [10]. There is a dearth of literature on prevalence of hyperglycemia among HIV infected children on DTG based regimen. Nonetheless, in an adult study in Uganda reported a prevalence of 12.8% of hyperglycemia among people living with HIV on dolutegravir based regimen [6]. In addition, Mengistu and colleagues in Ethiopia, reported a lower prevalence compared to ours, which was associated with participant’s age [14]. The reasons for the higher prevalences of hyperglycemia in these children compared with the findings from adult studies are not clear.

The prevalence of hyperglycemia in this study was based on Glycated hemoglobin A1c results in combination with random blood sugar tests. HbA1c determines the blood sugar levels in the last 120 days [15]. While, RBS provides a snapshot of the blood glucose, which might under or overestimate the levels. In this study only 6.6% of the cases were diagnosed with random blood sugar. A similar finding was reported by Akello et al., in children living with HIV on DTG based therapy [10].

Dolutegravir interferes with magnesium at the cellular level, causing hypomagnesemia, which results in reduced insulin secretion from the pancreas and resistance at the receptors (GLUT 4) on the target tissues; hence, hyperglycemia [16].

Only 5.9% of the study participants had a known baseline blood sugar before being initiated on dolutegravir based antiretroviral treatment. A risk-based baseline glucose assessment is recommended for patients living with HIV prior initiation on DTG based treatment. However, this is not routinely done for HIV infected children, which creates a concern of whether some of these children might have had undiagnosed prediabetes/diabetes mellitus or it is purely a consequence of DTG combined treatment.

**Factors associated with hyperglycemia:** Child’s age above 13 years and red meat consumption were independently associated with hyperglycemia among the HIV infected children on dolutegravir in Kabale district. We did not find studies describing child’s age and red meat consumption as predictors of hyperglycemia among HIV infected children on DTG. However, a study by Han et al., 2022, on HIV seronegative children, reported child’s age to be significantly associated with hyperglycemia/prediabetes [17]. This might be related with interaction between dolutegravir and hormonal changes during growth spurt at this age that can increase the risk of insulin resistance and hyperglycemia [18].

Red meat consumption was also independently associated with hyperglycemia among HIV infected children on dolutegravir. We found no published study on association between red meat consumption and hyperglycemia among HIV infected children on dolutegravir. However, several studies of non-HIV infected adults, found that consumption of red meat was a significant predictor of prediabetes and type 2 diabetes mellitus [19,20]. The mechanism of red meat consumption and its role in blood glucose metabolism disorders might involve high saturated fat content, heme iron, and nitrates resulting into impaired pancreatic beta cell destruction and insulin resistance [21–23].

Socioeconomic and lifestyle factors might also have a role in hyperglycemia among these children living with HIV on dolutegravir-based treatment. Nonetheless, these were not significant in the multivariable analysis; children in urban residences were more likely to develop hyperglycemia. At the same time, those with peasant caretakers had lower odds of increased blood sugar levels. According to a study in China, 3.3% of children living in urban residences had elevated fasting blood sugar levels [24]. Urbanization is associated with reduced physical activity, overweight and obesity with consequences of metabolic disorders including hyperglycemia [25].

## Limitations

A cross-sectional study design was used which is unable to determine the causal relationship between the outcome and predictor variables. The children were not assessed for anemia, which might underestimate HbA1c results.

## Conclusion

One in every five children living with HIV on dolutegravir in Kabale district, southwestern Uganda, had hyperglycemia and 24% of these had diabetes mellitus. Teenagers and those who had taken red meat in the last 24 hours were more likely to have hyperglycemia. Lifestyle modification and regular blood sugar monitoring should be emphasized in these children.
